# Does access to a colorectal cancer screening website and/or a nurse-managed telephone help line provided to patients by their family physician increase fecal occult blood test uptake?: A pragmatic cluster randomized controlled trial study protocol

**DOI:** 10.1186/1471-2407-12-182

**Published:** 2012-05-17

**Authors:** Kathleen Clouston, Alan Katz, Patricia J Martens, Jeff Sisler, Donna Turner, Michelle Lobchuk, Susan McClement

**Affiliations:** 1 Department of Family Medicine Research, Faculty of Medicine, University of Manitoba, P228-770, Bannatyne Ave, Winnipeg, MB R3E 0W3, Canada; 2 Departments of Family Medicine (Research Director) and Community Health Sciences, Faculty of Medicine, University of Manitoba, P228-770 Bannatyne Ave, Winnipeg, MB R3E 0W3, Canada; 3 Professor, Department of Community Health Sciences, Manitoba Centre for Health Policy (Director), Faculty of Medicine, University of Manitoba, Manitoba, Canada; 4 Director, Primary Care Oncology Program, CancerCare Manitoba; Associate Professor, Departments of Family Medicine and Internal Medicine, University of Manitoba; Chair, Canadian Association of General Practitioners in Oncology, Manitoba, Canada; 5 Population Oncology, CancerCare Manitoba, Department of Community Health Sciences, Faculty of Medicine, University of Manitoba, Manitoba, Canada; 6 Associate Professor, Faculty of Nursing, University of Manitoba, Manitoba, Canada; 7 Professor, Faculty of Nursing, University of Manitoba; Research Associate, Manitoba Palliative Care, Research Unit, CancerCare Manitoba, Manitoba, Canada

**Keywords:** Colorectal cancer screening, Fecal occult blood test, Community-based Primary healthcare research, Community-based clinical practice, Family physicians, Patient decision aid, Pragmatic, Cluster randomized controlled trial, Integrated knowledge translation, Knowledge exchange

## Abstract

**Background:**

Fecal occult blood test screening in Canada is sub-optimal. Family physicians play a central role in screening and are limited by the time constraints of clinical practice. Patients face multiple barriers that further reduce completion rates. Tools that support family physicians in providing their patients with colorectal cancer information and that support uptake may prove useful. The primary objective of the study is to evaluate the efficacy of a patient decision aid (nurse-managed telephone support line and/or colorectal cancer screening website) distributed by community-based family physicians, in improving colorectal cancer screening rates. Secondary objectives include evaluation of (dis)incentives to patient FOBT uptake and internet use among 50 to 74 year old males and females for health-related questions. Challenges faced by family physicians in engaging in collaborative partnerships with primary healthcare researchers will be documented.

**Methods/design:**

A pragmatic, two-arm, randomized cluster controlled trial conducted in 22 community-based family practice clinics (36 clusters) with 76 fee-for-service family physicians in Winnipeg, Manitoba, Canada. Each physician will enroll 30 patients attending their periodic health examination and at average risk for colorectal cancer. All physicians will follow their standard clinical practice for screening. Intervention group physicians will provide a fridge magnet to each patient that contains information facilitating access to the study-specific colorectal cancer screening decision aids (telephone help-line and website). The primary endpoint is patient fecal occult blood test completion rate after four months (intention to treat model). Multi-level analysis will include clinic, physician and patient level variables. Patient Personal Health Identification Numbers will be collected from those providing consent to facilitate analysis of repeat screening behavior. Secondary outcome data will be obtained through the Clinic Characterization Form, Patient Tracking Form, In-Clinic Patient Survey, Post-Study Follow-Up Patient Survey, and Family Physician Survey. Study protocol approved by The University of Manitoba Health Research Ethics Board.

**Discussion:**

The study intervention has the potential to increase patient fecal occult blood test uptake, decrease colorectal cancer mortality and morbidity, and improve the health of Manitobans. If utilization of the website and/or telephone support line result in clinically significant increases in colorectal cancer screening uptake, changes in screening at the policy- and system-level may be warranted.

**Trial registration:**

Clinical trials.gov identifier NCT01026753

## Background

The CIHR/CancerCare Manitoba Team in Primary Care Oncology Research is based in Winnipeg, Manitoba, Canada and is one of five Canadian Institutes of Health Research (CIHR) Primary Care Oncology New Emerging Team (PCO-NET) grant recipients (Additional file 1). The focus of our research is to investigate ways of supporting community-based family physicians in providing colorectal cancer services throughout the care continuum. We are a multi-disciplinary team of stakeholders and knowledge users including primary healthcare researchers, practitioners, policy and decision makers, and patients. The purpose of this team is to develop research initiatives that support evidence-based primary care oncology clinical practice, thus providing access to high quality cancer care.

Each week, approximately 430 Canadians will be diagnosed with colorectal cancer (CRC) and 175 will die of the disease, making it the second leading cause of cancer related death in Canada [1]. In Manitoba, CRC is the third most commonly diagnosed cancer (with an estimated 810 new cases in 2011) and the second leading cause of death from cancer in men and women combined (with an estimated 320 deaths in 2011) [1]. Colorectal cancer (CRC) screening rates for average risk individuals are sub-optimal in across Canada and in Manitoba [2]. Self-reported data from Manitobans indicates that approximately 38% of eligible individuals have completed an FOBT in the past two years [3]. This figure is close to that reported by Statistics Canada (2009) indicating FOBT screening rates for Manitobans of 41.9% [1]. Increased screening would reduce the physical, emotional and economic burden of CRC. It has been estimated that if approximately 70% of Canadians aged 50 to 74 completed an FOBT every two years, followed up by colonoscopy for positive FOBTs, the CRC mortality rate could be reduced by 17% [4-6].

Ninety three percent of all cases of CRC occur in those over the age of 50 and the incidence rate of the disease is expected to increase by 20% by 2025 [1,7]. CRC is a silent disease with overt symptoms not usually occurring until an advanced stage [8]. Early detection and treatment is vital in reducing the disease impact because survival decreases with increasing stage of disease at diagnosis [9]. Randomized control trials have shown that screening using the FOBT can reduce mortality from CRC by 25% [10]. A number of Canadian organizations have outlined CRC screening recommendations emphasizing FOBT at least every two years for average risk men and women between 50 to 74 years of age [1,11-14].

In Manitoba, patients may access FOBT screening through one of two routes: (1) a population-based screening program (ColonCheck) in Manitoba promotes screening through direct mailing, distribution at mammography clinics, the Mobile Breast Screening Program, publicity campaigns) but is not yet available to all Manitobans, and (2) Family physicians (FPs) which are the second access point to screening with an FOBT. The complexities of clinical practice and time constraints of the FP impact on the time available to adequately address all health concerns during the periodic health examination, especially prevention and screening [15]. Today, family physicians are responsible for caring for patients with increasingly complex multiple morbidities [16,17] and face challenges in addressing health promotion within the time constraints of clinical practice. Practitioners may also have insufficient time to access current evidence-based research findings and guidelines to support them in their CRC screening efforts [18], making knowledge translation activities between researchers and practitioners a vital component of the protocol objectives in the context of primary care research.

Tools that support family physicians in providing their patients with CRC screening information and support them in completing the test may prove useful in improving FOBT uptake among patients. In clinical practice, patients are usually given the FOBT through a laboratory requisition at their periodic health examination (PHE), provided the FOBT card from the physician’s support staff, or given it directly by their family physician and instructed to return it to the laboratory after they have completed it (unpublished observations; KC).

The first point of contact for a majority of patients seeking healthcare is with a community-based family physician. Family physicians play a key role in quality healthcare delivery, including CRC screening [19]. Manitobans depend on their family physicians to inform them of the need for FOBT testing in the prevention of CRC [8]. Physicians estimate it takes approximately four minutes to do a good job of explaining CRC and relevant screening options [20]. This represents between 27 and 40% of the total time available during the periodic health examination. In 2005, a study by Stokamer et al. demonstrated an increase in FOBT compliance from 51.3% to 65.9% using intensive one-on-one CRC education by registered nurses that took an additional 4.6 minutes beyond the time of standard CRC screening patient education (consisting of written instructions for the FOBT and verbal instruction to return the completed test within two weeks) [20]. Family physicians require supportive tools to facilitate FOBT recommendation and compliance among their patients to improve overall FOBT screening rates. Research is required to identify innovative methods of supporting family physicians in their role in CRC screening. Developing strategies aimed at supporting both the primary care provider as well as their patients has the potential to lead to substantial improvements in FOBT screening rates. Patient education about CRC and the importance of screening has been demonstrated to improve patient knowledge and compliance with FOBT [20,21]. Substantial improvements in FOBT completion rates have been demonstrated by studies that emphasized the importance of the FOBT, increased patient confidence in their ability to complete the FOBT, taught patients how to do the test, and provided time for patients to ask and receive answers to their individual questions about CRC and the FOBT [20]. Intervention strategies resulting in higher completion rates commonly involve one-on-one patient contact in clinics by registered nurses which is costly, time consuming [20], and uncommon in most community-based fee-for-service clinical practices in Manitoba. It may be more cost-effective to utilize a less expensive strategy, specifically a telephone support line managed by registered nurses. Another alternative may also involve access to a website with CRC information and FOBT assistance for patients. Although personalized emails from a physician reminding patients to undergo CRC screening and provided a link to a webpage with information about CRC did not improve FOBT compliance among patients [22], a multimedia educational computer program was demonstrated to be as effective as usual nurse counseling in educating patients and achieving adherence to FOBT screening [23].

Both patients and family physicians seem to be involved in a complex scenario resulting in sub-optimal colorectal cancer screening rates. There has been progress in patient awareness of colorectal cancer and the importance of screening [2]. A number of unique barriers to FOBT screening remain which lead to poor patient compliance [3]. In 2008, a colorectal cancer screening survey of 2,230 Manitobans aged 50 to 74 years demonstrated that only 26% had an FOBT within the previous year [3]. Factors identified by patients in the survey as contributing to low uptake among those eligible for CRC screening included: i) lack of knowledge about CRC and understanding of the significance and role of CRC screening in preventing and detecting the disease [2]; ii) lack of familiarity with the purpose of the FOBT; (iii) barriers associated with the test itself including the instructions for performing the FOBT [24,25] and iv) perception that the required collection of stool samples is an unpleasant task [26]. Additional reasons given by patients for not completing the FOBT included that: i) they didn’t think it was really needed or necessary (19%); ii) they thought the test sounded complicated (3%); iii) they didn’t really understand the test and why they should do it; iv) they did not want to handle stool (3%); and v) they meant to but didn’t think about it or forgot (3%) [3]. The survey also revealed that of those provided with an FOBT, 10% did not complete it. Among the reasons for completing an FOBT test, 79% of Manitobans surveyed said that it was included as part of a routine physical check-up or screening. Among those who did not do the test, 75% said it was because their doctor did not suggest it. This highlights the impetus and objectives of the protocol, to address the constraints of clinical practice and support family physicians in initiating CRC screening with their patients while at the same time, promoting patient understanding of the disease and the desire to take action and complete the test.

This protocol addresses the importance of promoting understanding of the FOBT among patients as well as their level of awareness, knowledge and education about CRC and screening practices. At the same time, it addresses the time constrains encountered by family physicians in adequately addressing CRC screening with their patients.

The patient decision aid used in this protocol is based on the Health Belief Model in that patient health prevention behaviors are largely determined by perceived susceptibility and seriousness of a health threat or personal risk [21], the patients’ consideration of benefits and barriers to action, including adequacy of information to cue action and self-efficacy or confidence in the ability to successfully take action [21]. Patient barriers to FOBT compliance are consistent with the Health Belief Model as they include limited accurate knowledge and understanding of CRC and screening tests for CRC, low perception of personal risk for CRC in the average-risk population, inconvenience, aversion to stool testing, lack of confidence in the ability to do the FOBT, the perception that the FOBT is time consuming, and fear of the consequences of screening [18,20].

In 2009, 66% of Canadians 45 years and older went online and represent an age group that is typically slower to adapt to and use the internet [27]. In 2010, 77.1% of Canadians had home internet access compared to 73% of Manitobans. Among those using the internet from home, approximately 75% went online every day in a typical month and 70% searched for medical or health related information online [28]. Seventy-four percent of females searched for information about health or medical conditions compared to 66.7 of men. In 2009, 71.6% of at home internet users were between 35–54 years of age compared to 69.1% of those 55–64 years, and 65.9% of those 65 years and older [29]. In 2010, 80% of individuals between the ages 45–64 used the internet compared to approximately 51% aged 65–74 and 27% aged 75 years and older. Household income quartile as well as urban versus rural community internet access are factors influencing internet use [27]. The role of the internet in assisting with and supporting positive health promotion behaviors in Manitoban’s age 50–74 years of age is currently unknown. Findings from the study outlined by this protocol will illuminate whether the internet is a possible medium for consideration as a means to support community-based family physicians in improving colorectal cancer screening of their patients.

## Methods/design

### Setting and participants

**Figure 1:**
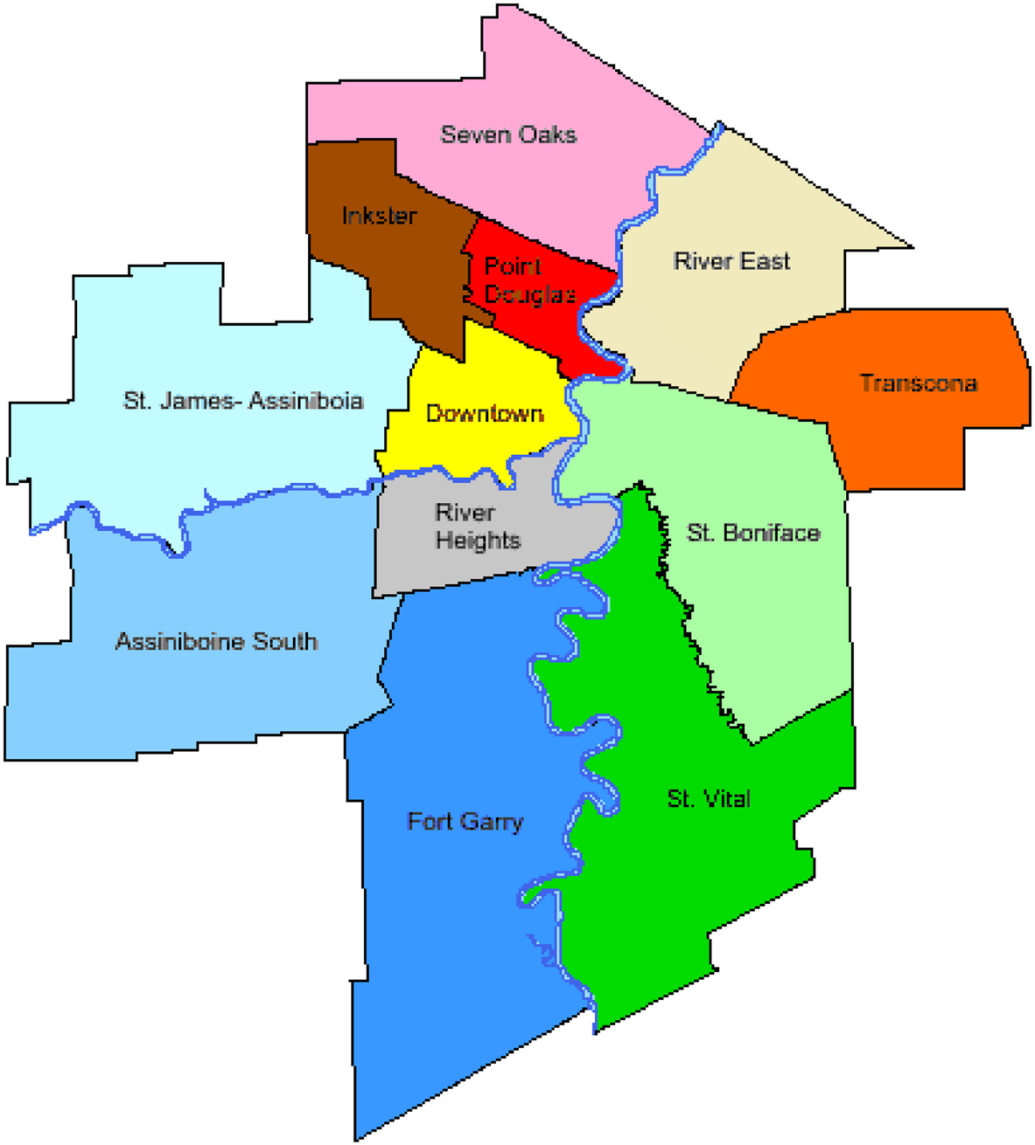
**The 12 community areas of Winnipeg, Manitoba, Canada. **The seven community areas represented in the study protocol include St. James-Assiniboia, Assiniboine South, Downtown, River Heights, St. Boniface, St. Vital, and Fort Garry. These represent those areas not yet involved in the province’s colorectal cancer screening program.

Family physicians will be recruited over a six month period from seven community areas (Figure 1) within the city of Winnipeg, Manitoba, Canada. Winnipeg is the capital city of the province with a population of approximately 685,000 (half the population of the province). In order to be eligible for the study, FPs must be in regular family practice (full or part-time), fee-for-service, solo or group practice and may be a member of the Physician Integrated Network and/or Uniting Primary Care and Oncology Network (Table 1). Once a family physician/medical clinic has agreed to participate in the study, a Family Physician/Clinic Characterization Form (Additional file 2) will be completed to document the routine care provided by each family physician in regards to CRC screening practices with the FOBT, whether they utilized an electronic medical record, have an on-site laboratory, and to document any additional information necessary to ensure minimal intrusiveness of the study protocol in the daily functioning of the clinical practice. Each family physician will enroll 30–35 patients who are: (1) attending their periodic health exam; (2) considered at average risk of developing colorectal cancer (Table 2); and (3) provide informed consent to participate. A patient tracking form (Additional file 3) will be filled out by the family physician for each patient enrolled.

**Table 1:** Description of stratification criteria Used in randomizing medical clusters to study groups

**Stratification criteria**	**Description**
**Physician Integrated Network (PIN) **[30]	A Manitoba Health primary care renewal initiative that focuses on fee-for-service (FFS) physician groups.
	Its goal is to facilitate systemic improvements in the delivery of primary care.
	Objectives:
	● To improve access to primary care
	● To improve Primary Care Providers’ access to and use of information
	● To improve the work life for all primary care providers
	● To demonstrate high quality primary care with a specific focus on Chronic Disease Management
**Uniting Primary Care and Oncology Network (UPCON)**[31]	A collaborative partnership between Winnipeg family practice clinics/primary health care centres and CancerCare Manitoba (CCMB) that is dedicated to promoting and supporting the shared care of the cancer patient.

**Table 2:** Patient eligibility criteria

**Inclusion criteria**	**Exclusion criteria**
Attending for a Periodic Health Examination	Walk-in patients
Males and females 50 to 74 years of age	Having had a colonoscopy in the last 10 years
No symptoms of colorectal cancer	Having had a flexible sigmoidoscopy within the last 5 years
No personal history of CRC, polyps, or diseases of the colon requiring monitoring by colonoscopy (Crohn’s Disease or Ulcerative Colitis)	Having a double contrast barium enema within the last 5 years
Have a first degree relative with CRC affected at age greater than 60	
Have two or more second degree relatives with polyps or cancer	

From our initial recruitment efforts, we determined that there were three methods by which patients receive an FOBT. Most FPs provide a laboratory requisition form to patients who are then given the test kit by laboratory staff. Alternatively, the FPs support staff may provide the FOBT kit directly to patients (with a requisition attached) or the FP may give the FOBT Kit directly to their patients (with a requisition attached). All FPs will continue with their current practice which will be documented. Whether kit distribution method affects patient FOBT uptake will be assessed. The rationale for customizing this portion of the study protocol is to optimize FP collaboration (recruitment) and to simulate “real world” clinical practice. Hence, the study findings will be relevant to practitioners and more likely used to make evidence-based decisions that are translated into practice.

### Sample size determination, randomization, and statistical analysis

Individual randomization of physicians within a group-practice and individual patients to either the control or intervention arm of the study is not feasible [32]. A cluster randomized design was chosen in which family physicians within a group practice were randomized to the same experimental group (control or intervention) and considered a cluster. Randomization of clusters will be conducted by a biostatistician using a computer-generated list and all of a physician’s patients will receive the same experimental treatment (control or intervention). Our protocol defines a multi-level analysis facilitating analysis of the intraclass correlation coefficient (ICC) for each primary group-level outcome (patients, FP, clinics/clusters) [32]. To ensure an even distribution of cluster-level characteristics between experimental groups, clusters will be randomized within strata based on membership in the Physician Integrated Network (PIN) and/or Uniting Primary Care Oncology Network (UPCON) [28]. These represent two quality improvement initiatives in Manitoba (Table 2). Clusters will be block randomized based on the number of collaborating FPs within a practice. An absolute increase in FOBT screening rates of 15% from the current 42% in Manitoba will be considered clinically significant [14]. Sample size was determined by a biostatistician using PASS (Power and Sample Size, 2002) software and a computer simulated ICC value of 0.6 [33] taking into consideration the clustered design of the trial [34]. This is a very conservative estimate of the minimum detectable effect size (MDEF) given the proposed sample size. Based on logistic regression a cluster size of 41 clusters each enrolling 30 to 35 patients (of which 80% are in the group that does not utilize the supplied intervention/magnet and 20% are in the group that does utilize the intervention/magnet; 1230 observations) will achieve 90% power at a 0.05 significance level to detect a change from the baseline value of 0.400 to 0.550 and corresponds to an odds ratio of 1.833. In the event that family physician collaboration/retention and/or patient recruitment proves to be more difficult than anticipated, a logistic regression with a cluster size of 28 (840 observations) achieves 80% power with the same outcomes.

Research has shown a majority of patients completing the FOBT do so within 4 to 6 weeks [20]. In order to allow adequate time to complete the FOBT and return it to the laboratory, patients will be given four months to do so. Those patients not returning their FOBT test within a period of four months will be scored as failing to complete the FOBT test.

Analysis of results will be based on the intention to treat (ITT) model. All Statistical analysis will be performed using SAS 9.2 ® software. Categorical variables will be compared using the Chi-square test. The time taken to return FOBT cards will be analyzed with Cox’ Proportional Hazards Regression analysis. Due to the clustered design, study outcomes will be measured at the level of the patient, family physician, and medical clinic/cluster. The analysis will utilize multi-level logistic regression that accounts for variance among patients with the same FP, FP within the same cluster, and among the clusters. The SAS® GLIMMIX procedure will be used to model patient FOBT completion rate (response variable). This will enable evaluation of the rate of patient FOBT compliance within medical clusters and individual FPs. Sources of variance included in the model will include cluster level (treatment, electronic medical record or paper charts, PIN and/or UPCON membership), family physician level (gender, solo/group practice), and patient level (age, gender, socioeconomic status, and previous FOBT screening history) variables as well as random variation.

A computerized randomization number list will be used to select 10 patients per FP to be contacted for a Post-Study Follow-Up Survey (Additional files 4 and 5) conducted by telephone. Only those patients providing permission to be contacted will be included. Those patients surveyed will be asked for permission to document their Personal Health Identification Number which will facilitate future studies conditional to Health Research Ethics Board (HREB) approval of analysis of repeat colorectal cancer screening behavior over time.

### Creating collaborative partnerships between community-based family physicians and primary healthcare researchers

The first step in creating the collaborative partnerships with Winnipeg family physicians will involve composing a list of medical clinics and community-based family physicians within the outlined community areas. Once defined, the study coordinator (KC) will make randomized cold-calls to each medical clinic to inform them of the study and delivery of the primary care provider (PCP) recruitment letter*.* The PCP recruitment letter briefly outlines the proposed research study, provides information on a small honorarium to acknowledge the efforts of the FP/clinics ($500.00 for 1 to 2 FPs; $1000.00 for 3 to 5 FPs; $1,500.00 for 6–9 FPs; and $2,000.00 for ≥10 FPs) and inquires about interest in collaborating on the study. Meetings will be arranged with interested FPs/clinics to outline the study in more detail and to determine collaboration. All methods used establish collaborative partnerships with FPs will be documented in an attempt to establish insights into those strategies that best support these efforts.

### Experimental treatment group

**Figure 2:**
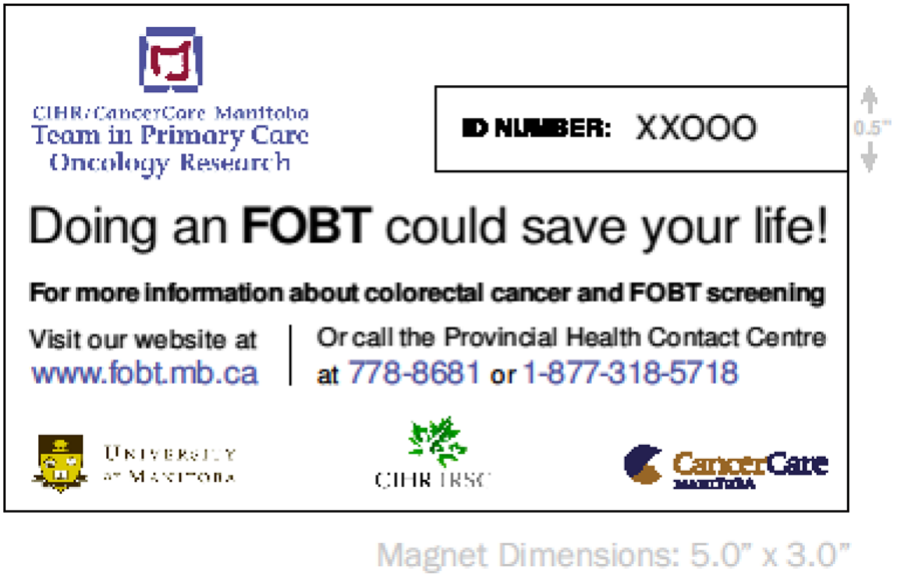
**Study magnet design. **Family physicians randomized to the intervention treatment group will provide each of their patients with a study refrigerator magnet (shown above) containing a unique seven character alpha-numeric study identification number that (1) facilitates access to the patient decision aids (a nurse-managed telephone support line and a website), (2) is linked to the Patient Tracking Form used by the family physician during the periodic health examination, and (3) linked to In-Clinic Patient Survey (Additional file 6). Family physicians will enroll between 30 to 35 patients who have provided their consent to participate in the study. The magnet was designed by the CIHR/CCMB Primary Care Oncology Research Team.

All FPs will follow their usual clinical practice for CRC screening with the FOBT during the patient periodic health examination. FPs in the intervention group will provide patients with access to the study specific colorectal cancer information and screening nurse-managed telephone support line and website by giving them a refrigerator magnet with the telephone number and website URL (Figure 2). Access to the telephone support line and/or website is patient initiated. All patients in both treatment groups will be assigned a study-specific, seven character, alpha-numeric identification number. For patients allocated to the intervention group, each patient’s magnet will be assigned the same study-specific identification number which will be used by patients in the experimental group to access the telephone and website patient aids, facilitate tracking patient use of the intervention support tools, and protect personal health information.

### The colorectal cancer information and screening telephone support line and website

The scientific literature and credible health resources [1,3,8,9,11-13] will be used to gather patient relevant colorectal cancer and screening information. Patient health education files will be generated based on this information to address patient’s most common questions and issues related to FOBT screening [3,8-10]. A survey conducted in Manitoba [3] and personal communications between the study coordinator (KC), family physicians and their support staff, and lab technicians will also inform the development of the patient decision aids. Website resource development was based on the same information in addition to feedback from CRC patients/survivors (n = 4), two small groups (n = 5–8) of average risk men and women, and the PCO-NET group during three quarterly workshops.

**Figure 3:**
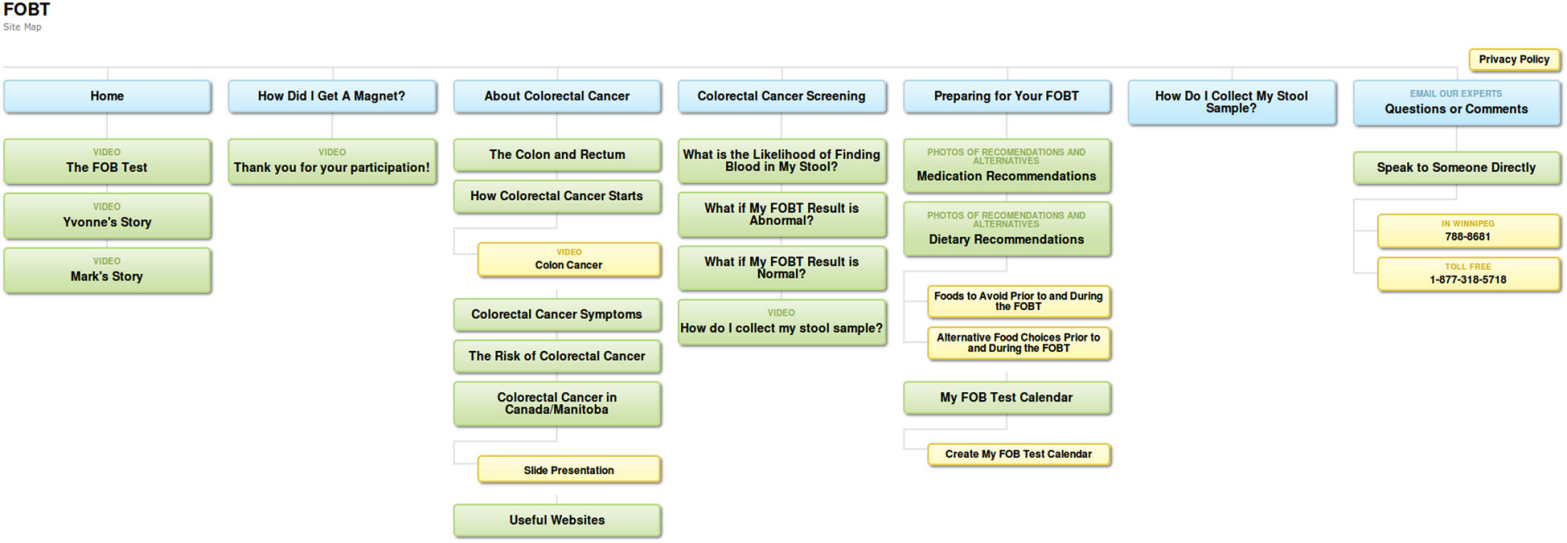
**Site Map for the colorectal cancer information and screening study website. **To obtain a website identification number and password to access to the study website, please contact Dr. Kathleen Clouston (kclousto@cc.umanitoba.ca).

Each patient will provide the registered nurse taking the call with their personal seven digit alphanumeric study identification number. Similarly, patients logging on to the website will be guided to enter their study identification number and create a personal password that allows them access to the site. Patients initiating a call to the telephone line will be able to ask questions which will be answered by a registered nurse using the health education files. If a patient asks a question to which there is no health education file/answer, the question will be documented by the registered nurse and the patient given the contact information of the study coordinator and/or provided the website address. Patients logging on to the website are able to view each webpage according to their specific questions and areas of interest (Figure 3 for website site map). Each page visited by each patient will be recorded. Patients will be able to email the study coordinator if they have further questions/comments. We will be able to track the number of calls made by each patient, the health education files accessed by each patient, the number of times they access the website and the website content/pages they visit, as well as any additional questions asked that were not contained in the health education files or website. Patient study identification number will correspond to the study identification number on the Patient Tracking Form which facilitates linking patient demographic information, FOBT completion status, and In-Clinic and Follow-Up Survey data with telephone and website usage.

### Description of the study protocol

**Figure 4:**
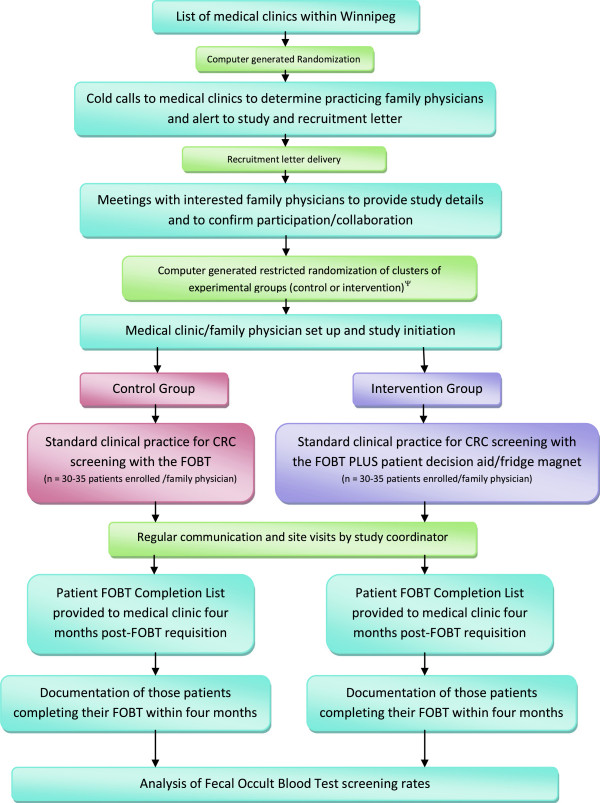
**Flow Diagram of study protocol for family physicians. **Ψ, strata based on membership in the Physician Integrated Network (PIN) and/or Uniting Primary Care and Oncology Network (UPCON); blocked based on the number of family physicians per cluster.

**Figure 5:**
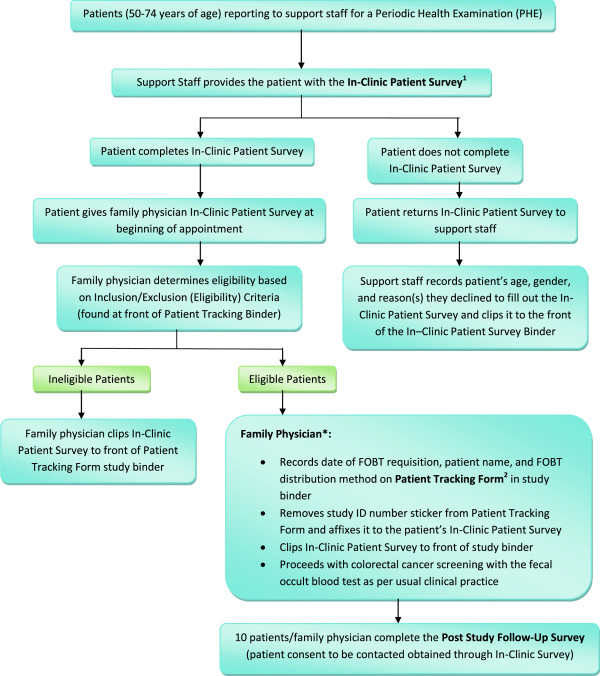
**Flow diagram of control group Study Protocol for family physicians and support staff.** * Family Physicians will complete the Family Physician Survey (Additional file 11) upon completion of study requirements. Please refer to text for further details.

**Figure 6:**
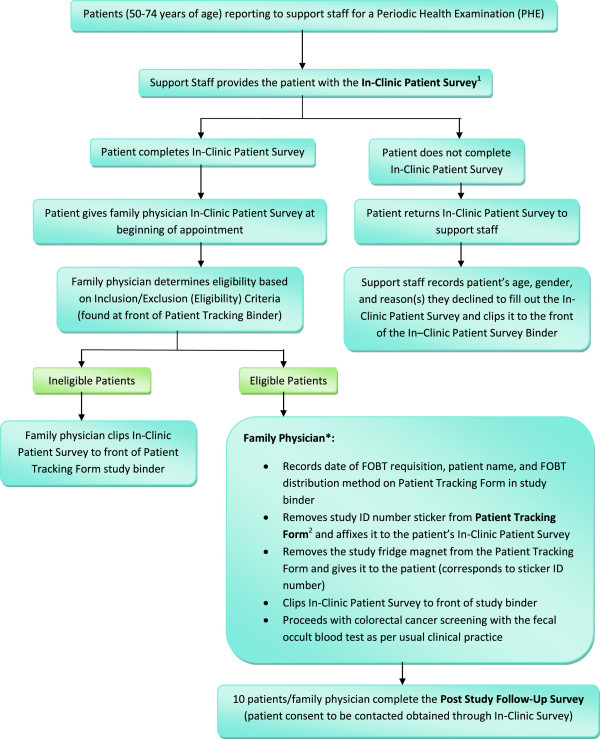
Flow diagram of intervention group study protocol for family physicians and support staff. * Family Physicians will complete the Family Physician Survey upon completion of study requirements. Please refer to text for further details.

This study protocol was approved by The University of Manitoba Health Research Ethics Board (Additional files 7, 8, 9 and 10) and is outlined in detail in Figures 4, 5, 6 and Tables 3–4. Table 5 provides an outline of and link to the study protocol forms and surveys along with a summary of the information obtained from each and the anticipated number of responses. Table 6 provides a description of the contents of the study set-up package. Upon arrival for their periodic health examination, eligible patients (Table 2) in both study groups will be asked by medical clinic support staff to complete the In-Clinic Patient Survey. This survey provides an explanation of the study and asks the patient to respond to a few general questions related to colorectal cancer screening, computer use for health-related questions, and permission to be contacted for the follow-up survey. It will function as the patient consent form. A business card will be attached to each In-Clinic Patient Survey providing the telephone numbers for the University of Manitoba Health Research Ethics Board and the Study Coordinator to facilitate patient contact as necessary. Patients declining to answer the survey return it to the clinic support staff who document that the patient declined to participate. Those patients completing the In-Clinic Survey will bring it with them and gave it to their FP during their periodic health examination. The FP will determined patient eligibility. All FPs conduct CRC screening as per their standard clinical practice. If a patient is ineligible, the FP will not enroll the patient. If the patient is eligible, the FP will enroll the patient by filling out a Patient Tracking Form. FPs will have a Patient Tracking Form Binder containing a Patient Tracking Form for each eligible patient in each of their evaluation rooms. Each Patient Tracking Form will have a removable sticker with the unique study identification number on it. The FP will remove and affixed this sticker to the patient’s In-Clinic Survey. Patients will be given four months to complete their FOBT. FPs in the intervention group will provide each eligible patient with a refrigerator magnet (paper clipped to the corresponding Patient Tracking Form). The protocol design allows us to determine how many patients provided with the magnet called the telephone support line as well as the number of times they called and their most common questions. It also allows us to determine which patients accessed the website, the pages they visited, and the information they were most interested in. Approximately four months after the last patient is enrolled, each FP will be provided with a list of their enrolled patients on which they will document the date of FOBT completion for those patients completing the test or an incomplete (for those not completing the FOBT). Patient FOBT results will not be collected. Using a computer generated randomization list, 10 patients per FP who agreed to be contacted for the Post-Study Follow-Up Survey will be contacted via telephone to complete the survey.

**Table 3:** Study protocol steps for control group

**Study protocol step**	**Description/details of study protocol step**
**STEP #1**	Patients between the ages 50–74 report to the reception desk/support staff for their Periodic Health Examination (PHE) or regular visit in which the family physician determines there is an opportunity for colorectal cancer screening (Fecal Occult Blood Test; FOBT).
**STEP #2**	Support staff provide patients meeting the above criteria with the In-Clinic Patient Survey (found in the study binder marked In-Clinic Patient Surveys) on a clipboard.
**STEP #3**	Patient decides whether they will fill out the In-Clinic Survey.
	If a patient declines to fill out the In-Clinic Survey, the support staff documents the patient’s age, gender, and reason(s) they declined to fill out survey (on the back of the In-Clinic Survey). Please clip the survey to the front of the study binder marked In-Clinic Patient Surveys.
	If a patient fills out the survey, they take it with them and give it to their family physician at the beginning of their appointment. The clipboard is returned to support staff so they are able to use it for the next patient.
**STEP #4**	The patient gives their In-Clinic Patient Survey to their family physician at the beginning of their appointment.
**STEP #5**	The family physician takes the In-Clinic Survey from the patient and determines if they are eligible for the study based on the inclusion/exclusion criteria found at the front of the family physicians study binder marked Patient Tracking Forms.
	If patients are ineligible, clip the In-Clinic Survey to the front of study binder marked Patient Tracking Forms.
	If patients are eligible:
	Turn to an unused Patient Tracking Form in the study binder. Record the date, the patient’s NAME, and mark the checkbox indicating the method of FOBT distribution to the patient.
	Remove the study ID number sticker from the Patient Tracking Form and place it on the patient’s In-Clinic Survey.
	Clip the In-Clinic Survey to the front of the Study Binder.
	Proceed with colorectal cancer screening with the Fecal Occult Blood Test (FOBT) as per usual clinical practice.
**STEP#6**	Study coordinator (Dr. Kathleen Clouston) will arrange a convenient date to collect forms and determine those completing their FOBT. In the event that you require more In-Clinic Surveys or have a question/comment/concern, please contact Dr. Clouston at **272**–**3086** or kclousto@cc.umanitoba.ca

**Table 4:** Study protocol steps for intervention group

**Study protocol step**	**Description/details of study protocol step**
**STEP #1**	Patients between the ages 50–74 report to the reception desk/support staff for their Periodic Health Examination (PHE) or regular visit in which the family physician determines there is an opportunity for colorectal cancer screening (Fecal Occult Blood Test; FOBT).
**STEP #2**	Support staff provides patients meeting the above criteria with the In-Clinic Patient Survey (found in the study binder marked In-Clinic Patient Surveys) on a clipboard.
**STEP #3**	Patient decides whether they will fill out the In-Clinic Survey.
	**If **a patient declines to fill out the In-Clinic Survey, the support staff documents the patient’s age, gender, and reason(s) they declined to fill out survey (on the back of the In-Clinic Survey). Please clip the survey to the front of the study binder marked In-Clinic Patient Surveys.
	**If **a patient fills out the survey, they take it with them and give it to their family physician at the beginning of their appointment. Clipboard is returned to support staff so they are able to use it for the next patient.
**STEP #4**	The patient gives their In-Clinic Patient Survey to their family physician at the beginning of their appointment.
**STEP #5**	The family physician takes the In-Clinic Survey from the patient and determines if they are eligible for the study based on the **inclusion/exclusion criteria found at the front of the family physicians study binder marked Patient Tracking Forms.**
	If patients are **ineligible**, clip the survey to the front of Study Binder
	If patients are **eligible**:
	Turn to an unused Patient Tracking Form in the Study Binder and record the **date,** the **patient’s NAME,** and mark the **checkbox** indicating the method of FOBT distribution to the patient.
	Remove the study ID number sticker from the Patient Tracking Form and place it on the In-Clinic Survey.Remove study magnet from Patient Tracking Form and give it to the patient. Clip the In-Clinic Survey to the front of the Study Binder.
	Proceed with colorectal cancer screening with the Fecal Occult Blood Test (FOBT) as per usual clinical practice.
**STEP#6**	Study coordinator (Dr. Kathleen Clouston) will arrange a convenient date to collect forms and determine those completing their FOBT. In the event that you require more In-Clinic Surveys or have a question/comment/concern, please contact Dr. Clouston at **272**–**3086** or **kclousto@cc.umanitoba.ca**

**Table 5:** Outline of study protocol forms and surveys and anticipated number of responses

**Study protocol form/survey**	**Individual completing form/survey**	**Information obtained**	**Anticipated number of responses**
Clinic Characterization Form^1^	Medical Cluster and Family Physician	Standard FOBT practice; Electronic medical record; on-site lab; solo/group practice	50 to 80
Family Physician Survey^2^	Family Physician	Experience with study protocol and value to clinical practice; facilitators and barriers to study collaboration; interest in future community-based primary healthcare research projects	50 to 80
FOBT Completion Status Form	Family Physician	Patient FOBT status four months post requisition)	50 to 80
Patient Tracking Form^3^	Family Physician	Date of FOBT requisition; Patient name; FOBT kit distribution method	1,500 to 2,400
In-Clinic Patient Survey^4^	Patient	Age, gender, postal code, Previous FOBT screening history, internet use for health related questions; exposure to advertisements related to FOBT screening; consent for Post-Study Follow-Up Survey	1,500 to 2,400
Post Study Follow-Up Survey (control)^5^	Patient (10 patients per family physician)	Colorectal cancer screening and FOBT experience with family physician; FOBT test instructions; computer access frequency of use; facilitators and barriers to completing the FOBT; Personal Health Information Number	250 to 400
Post Study Follow-Up Survey (intervention)^6^	Patient (10 patients per family physician)	Colorectal cancer screening and FOBT experience with family physician; FOBT test instructions; computer access frequency of use; facilitators and barriers to completing the FOBT; experience with and usefulness of patient decision aids (telephone-support line and website); Personal Health Information Number	250 to 400

^**1, 2, 3, 4, 5, 6**^ Additional files 2, 11, 3, 6, 4 and 5 for each form and survey.

**Table 6:** Description of study set-up package

**Study package delivered to each collaborating family physician**	**Description/details**
**In-Clinic Patient Survey Binder(s):**	Contents:
● thank you page for support staff	● In-Clinic Patient Surveys for support staff
● step-by-step outline of the study protocol,	● business card attached to each survey providing contact information for the study coordinator and the University of Manitoba Health Research Ethics Board (attached to clipboards and replaced when necessary)
● step-by-step flow diagram of the study protocol	
● patient eligibility criteria	
● contact information for the study coordinator	
**Clipboards with pens**	Two clipboards and pens per family physician
**Patient Tracking Form Binders:**	● Each family physician is provided with one Patient Tracking Form Binder per evaluation room (usually two)
● step-by-step outline of the study protocol	● the 30–35 Patient Tracking Forms are split between the these binders
● step-by-step flow diagram of the study protocol	
● patient eligibility criteria	● Each Patient Tracking Form corresponds to a specific patient with corresponding seven-digit alphanumeric study identification number
● contact information for the study coordinator	● family physician records the date of FOBT requisition, patient’s name, FOBT distribution method, removes the sticker from the form and attaches it to the corresponding patient’s In-Clinic Survey

The study coordinator will conduct site visits at regular intervals (approximately every two to three weeks) to facilitate regular communication, answer questions, and address issues with family physicians/support staff/medical clinics, build rapport and trust with partners, ensure successful study protocol implementation, and to pick up In-Clinic Surveys and Patient Tracking Forms.

### Primary outcome

The primary outcome of the study is the proportion of patients in each group who complete the FOBT test and returned it within four months. The proportion of patients in the intervention group who access the telephone support line and/or website will be determined. Of those who access the patient aids, the number of patients who complete their FOBT will be determined to facilitate evaluation of whether access to the patient aids by intervention group patients increased their rate of FOBT completion compared to the control group patients. Our findings will assist in determining whether the patient aids offered to the intervention group may be a useful tool to support FP in their CRC screening efforts and improve patient uptake of the test.

### Secondary outcomes

Secondary outcomes of the study include determining the most common questions of average-risk Manitoban patients’ related to CRC and FOBT screening and evaluating their experience with the telephone support line and/or website including helpfulness, number of times accessed, and preference for the telephone support line and/or website. Factors related to patient noncompliance will also be evaluated in post-study Follow-Up Survey. Each FP collaborating on the study was asked to complete a Family Physician Survey developed specifically for the study and will provide feedback from family physicians related to FOBT screening, their experience participating in this practice-based primary care research study, and the implementation/utilization of the patient aids in their clinical practice. All FPs will be provided with the study findings. In particular, FPs will be provided with their patient FOBT completion rates as well as those of their colleagues and the clinics collaborating in the study. All findings are confidential and reported anonymously. Costs of implementation of both the telephone support line and website will be evaluated.

## Discussion

Family physicians understand that colorectal cancer screening using the FOBT is effective in reducing colorectal cancer mortality and their patients are becoming more aware of the importance of screening. However, along the trajectory of colorectal cancer screening, a complex set of barriers exists for both family physicians and their patients.

If our findings demonstrate that utilization of the telephone support line and/or website result in clinically significant increases in FOBT compliance by average-risk Manitobans, changes in CRC screening at the policy- and system-level may be warranted. A strong and trusting family physician-patient relationship, combined with the patient intervention (education and decision aid) and public health programs to increase awareness of CRC and screening (ColonCheck Manitoba) has the potential to increase FOBT CRC screening rates, decrease CRC mortality and morbidity and improve the health of Manitobans.

It is expected that the patient-centered strategies outlined in the above protocol will support family physicians in improving patient understanding of CRC, the importance of FOBT screening in average-risk individuals as well as providing specific instructions on how to conduct the FOBT. Hence, it has the potential to lead to improved rates of patient FOBT compliance. Clinic, physician, and patient level variables associated with FOBT completion will be assessed for potential usefulness in planning strategies to target specific high-risk patient populations that require additional focus and supportive strategies for family physicians in Manitoba.

It is unknown what level of comfort/ease/acceptance Manitoban patients in the appropriate age range for CRC screening have with accessing/utilizing web-based health information and whether it has potential to be an effective venue through which FP could provide preventive health information to patients. This study will attempt to shed some light in this area.

Even if the outcome of the outlined study is negative, the findings will provide useful information regarding the limitations of the guaic based FOBT test in CRC screening and barriers to patient completion of the test. New potential research directions and study questions may also be generated.

Family physicians interested in collaborating in research face challenges including time pressures, minimal infrastructure to support the additional requirements of research, and lack of access to information about on-going research projects. In addition, their role primary healthcare research is often undervalued. We will document the factors involved in establishing the collaborative partnerships with the community-based family physicians in the outlined protocol.

This will allow us to contribute to the limited existing knowledge regarding successful partnerships among primary healthcare researchers and community-based family physicians as well as the unique requirements to support integrated knowledge translation and evidence-based decision making.

### Limitations

There are a few limitations of this study protocol which are outlined as follows:

(1) Inclusion of the In-Clinic Patient Survey in both treatment groups may raise patient awareness about CRC and screening sufficiently to affect/improve screening rates even without the intervention (magnet). To address this we will look at the CRC screening rates in the control group and compare them to historical rates.

(2) Those family physicians agreeing to participate in the study may have done so with awareness that their patients (screening process) have a relatively higher screening rate and degree of success with screening. FP declining to participate may have lower patient CRC screening rates and be concerned about participating, the perception of being evaluated, and how it reflects on their practice. It may be that these FP-patient populations have the most to gain/highest potential to benefit from the intervention. It may be that, in certain populations, the intervention increases screening rates more than in others.

(3) Some providers (PIN and UPCON) are involved in other quality improvement interventions. Therefore, these clinics may have a specific approach to screening that has already lead to improvements in patient CRC screening rates and functioning/practice may be more evidence-based compared to those that are not involved in these initiatives. In these groups, the study intervention may have minimal effect.

(4) Only fee-for service FPs are included. Therefore, the results may be applicable only to this type of provider.

(5) FP and patients represent an urban geographical area and therefore, results may not be generalizable to patient populations in rural areas.

(6) Disparity in accessing the website patient aid is a likely factor for individuals living in rural communities and those with a lower socioeconomic status. Those patients agreeing to the follow-up survey may be more apt to complete the FOBT. Therefore, feedback from those prone to fail to complete the test may be under represented.

### Benefits

There are a number of benefits of the study protocol which are outlined as follows:

(1) The pragmatic design and flexibility of the protocol for each medical practice ensured it reflected the “real world” clinical practice environment and its inherent variability. This approach will produce findings that are generalizable and relevant to community-based family practice and will increase translation and application of evidence-based findings into the clinical practice setting. The study was powered to detect an absolute increase in CRC screening rates of 15% by the intervention. This is a realistic and clinically significant improvement.

(2) The multi-disciplinary team working together on this project represent multiple stakeholder opinions and contributions (primary care providers, primary care researchers, policy makers, and patients). It also reflects a variety of providers/practitioners all of which enhance the pragmatic nature of the design and findings. Working collaboratively on this research project facilitated creation of new networking opportunities and partnerships that have the potential to support future primary healthcare research that inform evidence-based family practice.

(3) Findings will allow us to describe the degree of variability in screening rates among the medical clinics and practitioners involved in the study and, the rates of internet usage among the age category 50–74 for a health-related issue, and more specifically, CRC information and screening.

(4) Findings will provide valuable patient feedback about the FOBT and the common issues related to completion/non-compliance with the test.

(5) The study is highly representative of the community-based clinical practice environment and included a variety of FPs from a number of different areas in the city and practice settings.

## Abbreviations

CIHR: Canadian Institutes of Health Research; CPx: (annual physical); CRC: Colorectal Cancer; FOBT: Fecal occult blood test; FP: Family Physician; ICC: Intraclass Correlation Coefficient; ITT: Intention to Treat Principle/Model; PCO-NET: Primary Care Oncology New Emerging Team Research Group; PCP: Primary Care Provider; PIN: Physician Integrated Network; PHCC: Provincial Health Contact Centre; PHE: Periodic Health Exam (Annual physical); UPCON: Uniting Primary Care and Oncology/Urban Primary Care Oncology Network; WRHA: Winnipeg Regional health Authority.

## Competing interests

The authors declare that they have no competing interests.

## Authors' contributions

KC provided substantive intellectual contributions to the conceptualization, design and implementation, data acquisition, analysis and interpretation phases of the study protocol. KC wrote the first draft of the study protocol manuscript providing substantive intellectual content. All authors provided substantive intellectual contributions to the conceptualization, design, analysis, and interpretation phases of the study protocol. AK, PM, JS, DT, ML, and SM were involved in critically reviewing the manuscript for important intellectual content. The CIHR/CCMB PCO-NET group provided their intellectual contributions to the conceptualization, design, and interpretation phases of the study protocol. All authors gave final approval of the manuscript version submitted for publication. Dr. Cornelius Woelk is the designated CIHR/CCMB PCO-NET group representative. All authors read and approved the final manuscript.

## Pre-publication history

The pre-publication history for this paper can be accessed here:

http://www.biomedcentral.com/1471-2407/12/182/prepub

## Supplementary Material

Additional file 1 **Proof of Funding from a major granting body. **Letter from the Canadian Institutes of Health Research (CIHR) demonstrating proof of receipt of grant from a major funding body that supports the Theme Three Study (Trial Registration: clinicaltrials.gov identifier NCT01026753). Click here for file

Additional file 2 **Clinic Characterization Form. **Form utilized to obtain information about medical clinics collaborating on the study. Click here for file

Additional file 3 **Patient Tracking Form. **Form used by family physician to enroll eligible patients consenting to participate in the study and to record pertinent patient information. Click here for file

Additional file 4 **Post Study Follow-Up Survey (Control Group). **Survey completed by 10 consenting patients per family physician in the control group four months post FOBT requisition. Click here for file

Additional file 5 **Post Study Follow-Up Survey (Intervention Group). **Survey completed by 10 consenting patients per family physician in the intervention group four months post FOBT requisition. Click here for file

Additional file 6 **In-Clinic Patient Survey. **Consent form/Survey completed by patients enrolled in the study. Click here for file

Additional file 7 **Proof of Health Research Ethics Board (University of Manitoba) approval.** University of Manitoba Health Research Ethics Board approval of Theme Three research study entitled “Innovative Tools to Improve Colorectal Cancer Screening in Manitoba” (H2009:312); Trial Registration: clinicaltrials.gov identifier NCT01026753. Click here for file

Additional file 8 **Proof of Health Research Ethics Board (University of Manitoba) approval. **Final Health Research Ethics Board approval from the University of Manitoba Health Research Ethics Board. Approval of the theme three research study entitled “Innovative Tools to Improve Colorectal Cancer Screening in Manitoba” (H2009:312); Trial Registration: clinicaltrials.gov identifier NCT01026753. Click here for file

Additional file 9 **Proof of Health Research Ethics Board (University of Manitoba) annual approval (2010–2011). **University of Manitoba Health Research Ethics Board annual approval (2012–2011) of the theme three research study entitled “Innovative Tools to Improve Colorectal Cancer Screening in Manitoba” (H2009:312); Trial Registration: clinicaltrials.gov identifier NCT01026753. Click here for file

Additional file 10 **Proof of Health Research Ethics Board (University of Manitoba) annual approval (2011–2012). **University of Manitoba Health Research Ethics Board annual approval (2012–2011) of the theme three research study entitled “Innovative Tools to Improve Colorectal Cancer Screening in Manitoba” (H2009:312); Trial Registration: clinicaltrials.gov identifier NCT01026753. Click here for file

Additional file 11 **Family Physician Survey. **Survey completed by collaborating family physicians upon completion of study requirements. Click here for file
