# Supplementary material for: Does access to a colorectal cancer screening website and/or a nurse-managed telephone help line provided to patients by their family physician increase fecal occult blood test uptake?: A pragmatic cluster randomized controlled trial study protocol
Source: BMC Cancer. 2012 May 17;12:182. doi: 10.1186/1471-2407-12-182 (PMC3495851; doi:10.1186/1471-2407-12-182)
Supplement: Additional file 1 — Proof of Funding from a major granting body. Letter from the Canadian Institutes of Health Research (CIHR) demonstrating proof of receipt of grant from a major funding body that supports the Theme Three Study (Trial Registration: clinicaltrials.gov identifier NCT01026753). [file 1471-2407-12-182-S1.pdf]

Primary Care Provider (Family Physician)/Clinic Characteristics Form

Re: Innovative Tools to Improve Colorectal Cancer Screening Rates in Manitoba

Clinic Name: ____

PIN Site: Yes No

Address (postal code): ____

Telephone number(s): ____

____

FAX number: ____

Physician(s) Name(s): ____

____

____

____

Support Staff (contact): ____

____

Business Card(s) collected: ____

Paper-Based Patient Chart: Yes / No Electronic Medical Records: Yes / No

Laboratory used: ______ Trainor Laboratories

______ Central Medical Laboratories

______ Assiniboine Clinic Laboratory

Other: ____

Sample Laboratory Requisition collected (if appropriate): Yes / No

Innovative Tools to Improve Colorectal Cancer Screening Rates in Manitoba

Standard method of FOBT: ______ Checked off on Laboratory Requisition Form (given to the patient to take to the laboratory)

______ FOBT directly distributed to patient by PCP.

_______Study coordinator obtained an FOBT kit from those distributing FOBT kits to patients

Other (describe): ___________________________________

____

____

Does PCP inform patient they are requesting an FOBT test on Lab requisition?

Yes / No / Not Applicable

Does PCP inform patient they want the patient to do the FOBT? Yes / No

Typical communication(s) about the FOBT with patient (if any): ____________________

____

____

____
