# Supplementary material for: Does access to a colorectal cancer screening website and/or a nurse-managed telephone help line provided to patients by their family physician increase fecal occult blood test uptake?: A pragmatic cluster randomized controlled trial study protocol
Source: BMC Cancer. 2012 May 17;12:182. doi: 10.1186/1471-2407-12-182 (PMC3495851; doi:10.1186/1471-2407-12-182)
Supplement: Additional file 2 — Clinic Characterization Form. Form utilized to obtain information about medical clinics collaborating on the study. [file 1471-2407-12-182-S2.pdf]

Innovative Tools to Improve Colorectal Cancer Screening Rates in Manitoba

Family Physician Survey

Thank you for your participation in our study. Your collaboration in this study has been accredited by the College of Family Physicians of Canada and the Manitoba chapter for up to **8 Mainpro-M1 credits**.

This survey will take approximately 10 to 12 minutes to complete. It will help us understand your experience(s) collaborating on the study and inform future research endeavors relevant clinical practice.

General Practice Questions

1. During a periodic health examination (PHE or CPx), I encounter the following barriers in implementing colorectal cancer screening (**please check off all that apply**):

   ☐ Time constraints

   ☐ Lack of a reminder system

   ☐ Unsure of guidelines for FOBT screening

   ☐ Other ____

   ☐ None

2. I find it difficult to ensure my patients have completed their fecal occult blood test (FOBT) screening.

   Yes　　　　No　　(**If no, please go to question #4**)

3. A tracking system for patient FOBT screening would help me follow-up with patient FOBT screening status.

   Yes　　　　No

Research Collaboration Questions

4. I have participated in other research studies in the past?

   Yes No

5. Collaboration in the study was intrusive in the overall functioning of my clinical practice?

   1 2 3 4 5

   Not at All **Highly**

6. I found it difficult to implement the study protocol into the daily functioning of my clinical practice?

   1 2 3 4 5

   Not at All **Highly**

7. The barriers I encountered in implementing the study protocol into my clinical practice were: (**please check off all that apply**)

   ☐ Limited support staff resources to assist with study implementation

   ☐ Time constraints of family physician

   ☐ Practice change disrupting implementation

   ☐ Eligibility criteria for patient enrollment

   ☐ Lack of contact with study coordinator

   ☐ Other:____

8. My patients were receptive to filling out the In-Clinic Survey?

Yes No Unsure

9. Participating in the study caused me to reflect on my standard colorectal cancer screening practice(s)?

Yes No Unsure

10. Participating in the study caused me to change my standard colorectal cancer FOBT screening practice?

Yes No Unsure

11. Participating in the study will have a beneficial impact on my patients' health?

Yes No Unsure

12. Participating in the study will have a beneficial impact on my clinical practice?

Yes No Unsure

13. The study protocol caused my patients to ask more questions than usual about:

**Colorectal cancer**	Yes	No	Unsure
**Colorectal cancer screening**	Yes	No	Unsure
**Fecal occult blood test**	Yes	No	Unsure

14. I gained a valuable clinical learning experience by collaborating on this research study.

Agree Disagree Unsure

15. Factors that facilitated implementing the study protocol into my clinical practice were: (**please check off all that apply)**

- ☐ The small amount of paperwork to required to be completed by the physician
- ☐ Flexibility of the study protocol
- ☐ Good communication with my support staff
- ☐ Assistance from my support staff
- ☐ Contact with the study coordinator

16. I would like to be informed about future opportunities to collaborate on research projects?

Yes No Unsure (**If No, go to question # 18**)

17. Please indicate the best way(s) we can provide you with updates on future research opportunities: (**please indicate all that apply**)

- ☐ Email ____
- ☐ Support staff (name and telephone number)______________________

  ____
- ☐ FAX ____
- ☐ Telephone____
- ☐ Face-to-face meeting/contact

18. I have clinical practice questions I am interested in investigating?

Yes No Unsure

19. Have you heard about the Canadian Primary Care Sentinel Surveillance Network (CPCSSN)?

Yes No Unsure (**If NO, go to question #20**)

20. Would you like us to send you information the CPCSSN? The CPCSSN is a sub entity of the College of Family Physicians of Canada, whose mission is to collect and maintain national epidemiological surveillance data using EMRs to improve outcomes in primary health care and contribute to a stronger national knowledge base in the area of primary health care and chronic disease management.

Yes No

Any additional comments and feedback you would like to provide would be greatly appreciated!
