# Supplementary material for: Does access to a colorectal cancer screening website and/or a nurse-managed telephone help line provided to patients by their family physician increase fecal occult blood test uptake?: A pragmatic cluster randomized controlled trial study protocol
Source: BMC Cancer. 2012 May 17;12:182. doi: 10.1186/1471-2407-12-182 (PMC3495851; doi:10.1186/1471-2407-12-182)
Supplement: Additional file 3 — Patient Tracking Form. Form used by family physician to enroll eligible patients consenting to participate in the study and to record pertinent patient information. [file 1471-2407-12-182-S3.pdf]

CIHR/CancerCare Manitoba
Team in Primary Care
Oncology Research

Primary Care Provider Patient Tracking Form

CIHR/CancerCare Manitoba Team in Primary Care Oncology Research
Theme Three Research Study: Innovative Tools to Improve Colorectal Cancer Screening Rates in Manitoba

Primary Care Provider: ____________(filled out by study coordinator)________________________

Clinic (if applicable): ______________(filled out by study coordinator)________________________

Date (dd/mm/yy): ___(filled out by family physician)_______________________________

Patient Name: ______(filled out by family physician)_______________________________
(please print last name, first name)

☐ FOBT checked off on lab requisition

☐ FOBT given to patient by medical clinic support staff

☐ FOBT given to patient directly by family physician

Study Identification Number: ______(unique for each patient; filled out by study coordinator)___

Study Identification sticker:

Removable sticker containing the unique seven digit alpha numeric study identification number; removed by physician and placed onto the patient's In-Clinic Survey

(Please remove the sticker and affix it to the patient's In-Clinic Survey. Clip In- Clinic Survey to back of study binder.)

If you have any questions, please contact the study coordinator, Kathleen Clouston, at 272-3086 or kclousto@cc.umanitoba.ca
