# Supplementary material for: Does access to a colorectal cancer screening website and/or a nurse-managed telephone help line provided to patients by their family physician increase fecal occult blood test uptake?: A pragmatic cluster randomized controlled trial study protocol
Source: BMC Cancer. 2012 May 17;12:182. doi: 10.1186/1471-2407-12-182 (PMC3495851; doi:10.1186/1471-2407-12-182)
Supplement: Additional file 4 — Post Study Follow-Up Survey (Control Group). Survey completed by 10 consenting patients per family physician in the control group four months post FOBT requisition. [file 1471-2407-12-182-S4.pdf]

Patient-Specific Identification Number

In-Clinic Patient Survey

This survey is being conducted by the Faculty of Family Medicine researchers at the University of Manitoba as part of a research study looking at ways doctors can help their patients be screened for cancer of the colon and rectum (colorectal cancer). The at-home screening test, also called the Fecal Occult Blood Test (FOBT), is done in the privacy of your own home and detects small amounts of hidden blood in your stool (or poop) which may be a sign of colorectal cancer.

Answering the survey questions below will take approximately three minutes and pose no known risks to you. There may or may not be direct medical benefit to you from participating in this study. However, we hope the information learned from this study will benefit both family doctors and Manitobans in the future.

Your participation is voluntary. If you chose not to participate, it will not affect your medical care at this site. All information collected will be treated as confidential in accordance with the Personal Health Information Act of Manitoba.

Date: ____________________________ Postal Code: ______________________
(day/month/year)

Age: _______________________ Gender: ☐ Male ☐ Female

1. Have you ever done an FOBT (at-home screening test) before?

   ☐ Yes ☐ No ☐ Unsure

2. Have you done an FOBT (at-home screening test) within the last two years?

   ☐ Yes ☐ No ☐ Unsure

3. Has a doctor or other health care professional ever suggested you do an FOBT (at-home screening test)?

☐ Yes ☐ No ☐ Unsure

4. If so, did you do it?

☐ Yes ☐ No ☐ Unsure

5. Have you ever received an FOBT (at-home screening test) kit in the mail?

☐ Yes ☐ No ☐ Unsure

6. If so, did you do it?

☐ Yes ☐ No ☐ Unsure

7. Have you ever received an FOBT (at-home screening test) kit at a mammography clinic?

☐ Yes ☐ No ☐ Unsure

8. If so, did you do it?

☐ Yes ☐ No ☐ Unsure

9. Have you ever received an FOBT (at-home screening test) kit from a Mobile Breast Screening Program?

☐ Yes ☐ No ☐ Unsure

10. If so, did you do it?

☐ Yes ☐ No ☐ Unsure

11. Please indicate any/all of the following informational advertisements related to colorectal cancer and screening that you have seen lately:

- Newspaper advertisements ☐
- Television advertisements ☐
- Bus advertisements ☐
- Radio advertisements ☐
- Billboard advertisements ☐
- Websites (Internet) ☐
- Other (please specify) ____________________________

12. Have you ever used the internet to help you with a health-related question/issue?

☐ Yes ☐ No

13. Would you be willing to participate in a follow-up survey about this study within the next 4 months?

☐ Yes ☐ No

Name:____
(Please Print; first name, last name)

Telephone Number:____
(Please include area code if possible)

For Office Use Only

Age: ____________ years old
(please fill in patient's age)

Gender: male / female
(please circle the appropriate gender)

Reason(s) patient did not fill out the In-Clinic Survey:

____

____

____

____

____

____

Patient declined to answer: ☐
