# Supplementary material for: Does access to a colorectal cancer screening website and/or a nurse-managed telephone help line provided to patients by their family physician increase fecal occult blood test uptake?: A pragmatic cluster randomized controlled trial study protocol
Source: BMC Cancer. 2012 May 17;12:182. doi: 10.1186/1471-2407-12-182 (PMC3495851; doi:10.1186/1471-2407-12-182)
Supplement: Additional file 6 — In-Clinic Patient Survey. Consent form/Survey completed by patients enrolled in the study. [file 1471-2407-12-182-S6.pdf]

Post Study Follow-Up Patient Survey (Intervention Group)

Innovative Tools to Improve Colorectal Cancer Screening Rates in Manitoba

At your doctor's office a few months ago, you completed a survey about colorectal cancer screening and the fecal occult blood test (FOBT). At that time, you agreed to be contacted for a follow-up survey. We are asking you to complete the follow-up questions by providing the answer that best describes your experience. On behalf of the Department of Family Medicine we thank you for your time and participation!

Date: ______________________________
(day/month/year)

1. During the appointment with your health care provider in which you were asked to do a fecal occult blood test (FOBT), did you receive information about your risk of developing colorectal cancer?

   Yes No Unsure

2. During the appointment with your health care provider in which you were asked to do a fecal occult blood test (FOBT), did you receive information about colorectal cancer screening from your health care provider?

   Yes No (go to question number 4) Unsure (go to question number 4)

3. If you were provided with colorectal cancer and/or screening information, who provided it to you?

   - ☐ Doctor
   - ☐ Nurse
   - ☐ Other (please specify):___________________________________

4. Who gave you the fecal occult blood test (FOBT) kit?

☐ A lab technician

☐ Directly from my Family Physician/ Physician Assistant/Nurse Practitioner

☐ Physician's support staff

5. Other than the written instructions that came with the fecal occult blood test (FOBT) kit, did you receive any instruction on how to conduct the fecal occult blood test (FOBT)?

Yes No (go to question number 7) Unsure (go to question number 7)

6. If so, who provided you with information on how to conduct the FOBT?

☐ Doctor

☐ Nurse

☐ Lab Technician

☐ Physician's support staff

☐ Other (please specify):____________________________________

7. Did your doctor give you a refrigerator magnet containing a telephone number and website address to use to help you complete your fecal occult blood test (FOBT)?

Yes No Unsure

8. When you received your FOBT fecal occult blood test (FOBT), did you have a clear understanding of why you should do it?

Yes No Unsure

9. Did you have questions about the need for the test that were not addressed by your family physician?

Yes No Unsure

10. Did you complete your fecal occult blood test (FOBT)?

Yes (omit question numbers 21, 22, 23, 24, and 25) No Unsure

11. Were the written instructions that came with the fecal occult blood test (FOBT) sufficient to assist you in completing the fecal occult blood test (FOBT)?

Yes No Unsure

12. Did you call your medical clinic for information on colorectal cancer and/or instructions on how to conduct the fecal occult blood test (FOBT)?

Yes No Unsure

13. Did you call the laboratory for information on colorectal cancer and/or instructions on how to conduct the fecal occult blood test (FOBT)?

Yes No Unsure

14. Did you call the telephone number on the magnet for information on colorectal cancer, colorectal cancer screening and/or instructions on how to conduct the fecal occult blood test (FOBT)?

Yes No Unsure (**IF No or unsure**, please go to question number 16)

15. If so, was it useful in helping you complete your fecal occult blood test (FOBT)?

Yes No Unsure

16. Did you visit the website address on the magnet for information on colorectal cancer, colorectal cancer screening and/or instructions on how to conduct the fecal occult blood test (FOBT)?

Yes No Unsure (**IF NO or unsure**, please go to question number 18)

17. If so, was it useful in helping you complete your fecal occult blood test (FOBT)?

Yes No Unsure

18. Did you use any websites other than the magnet website to help you understand colorectal cancer, screening and/or how to conduct the fecal occult blood test (FOBT)?

Yes No Unsure

19. Did the information/assistance provided by using the telephone number affect your decision to complete the fecal occult blood test (FOBT)?

Yes No Unsure

20. Did the information/assistance provided by using the magnet website affect your decision to complete the fecal occult blood test (FOBT)?

Yes No Unsure

21. If you did not do the fecal occult blood test (FOBT), please indicate the factors you believe prevented you from doing it (please indicate all that apply)?

- ☐ Medication restrictions
- ☐ Dietary restrictions
- ☐ Dealing with feces (poop) an unpleasant task
- ☐ Uncertainty about how to do the test
- ☐ Not confident I could complete the test
- ☐ I felt the test was unnecessary
- ☐ Meant to do it but forgot

22. If you did not do the fecal occult blood test (FOBT), would additional information about colorectal cancer have made a difference for you in completing the test?

Yes No Unsure (**If YES**, please go to question number 23)

23. Please specify below what additional information about colorectal cancer and/or screening would have made a difference for you in completing the test:

____

____

____

____

____

____

____

____

24. If you did not do the fecal occult blood test (FOBT), would additional support related to how to do the fecal occult blood test (FOBT) have made a difference for you in completing the test?

Yes No Unsure (**If YES**, please go to question number 25)

25. Please specify below what additional information about how to do the fecal occult blood test (FOBT) would have made a difference for you in completing the test:

____

____

____

____

____

____

____

____

26. Do you use a toilet bowl cleaner? Yes No

27. Do you use any of the following (non-steroidal anti-inflammatory) drugs:

a.	Aspirin	Yes	No	amount per day: (optional)
b.	ibuprofen (Motrin or Advil)	Yes	No	amount per day: (optional)
c.	naproxen (Naprosyn, Aleve)	Yes	No	amount per day: (optional)
d.	celecoxib (Clebrex)	Yes	No	amount per day: (optional)
e.	indomethacin (Indocin)	Yes	No	amount per day: (optional)
f.	diclofenac (Voltaren)	Yes	No	amount per day: (optional)

28. Approximately how many oranges do you eat per week? ____________

29. Approximately how many grapefruit do you eat per week? ____________

30. If one serving of fruit juice is ½ of a cup, how many servings of juice do you "typically" consume in a day?_______________, in a week? ___________________

31. Do you take a vitamin C supplement? Yes No

32. Do you take a multi-vitamin-mineral supplement? Yes No

33. If a serving of vegetables is the amount that would fit into your hand:

   a. Approximately how many servings of broccoli do you consume in a "typical" week? ____________

   b. Approximately how many servings of cauliflower do you consume in a "typical" week? ____________

34. If red meat includes meat such as beef, lamb, moose, venison and bison (not chicken, fish or pork), how many times in a "typical" week do you eat red meat?

35. In a "typical" week, do you eat:

a. Cantaloupe	Yes	No	Decline to answer
b. Raw turnips	Yes	No	Decline to answer
c. Red radishes	Yes	No	Decline to answer
d. Parsnip	Yes	No	Decline to answer
e. Horseradish	Yes	No	Decline to answer

36. As part of this study, would you be willing to provide your personal health identification number which may be used at a later date, along with medical databases, to look at factors affecting colorectal cancer screening rates in Manitoba? Your personal health identification number or PHIN is the 9 digit number on your Manitoba health card. All information collected will be treated as confidential in accordance with the Personal Health Information Act of Manitoba. Your name and other identifying information will be removed from the information collected so as to ensure your safety and confidentiality.

   a. Yes PHIN: ________________________
   b. No
