# Supplementary material for: Does access to a colorectal cancer screening website and/or a nurse-managed telephone help line provided to patients by their family physician increase fecal occult blood test uptake?: A pragmatic cluster randomized controlled trial study protocol
Source: BMC Cancer. 2012 May 17;12:182. doi: 10.1186/1471-2407-12-182 (PMC3495851; doi:10.1186/1471-2407-12-182)
Supplement: Additional file 9 — Proof of Health Research Ethics Board (University of Manitoba) annual approval (2010–2011). University of Manitoba Health Research Ethics Board annual approval (2012–2011) of the theme three research study entitled “Innovative Tools to Improve Colorectal Cancer Screening in Manitoba” (H2009:312); Trial Registration: clinicaltrials.gov identifier NCT01026753. [file 1471-2407-12-182-S9.pdf]

UNIVERSITY OF MANITOBA

BANNATYNE CAMPUS
Research Ethics Boards

P126-770 Bannatyne Avenue
Winnipeg, Manitoba
Canada R3E 0W3
Tel: (204) 789-3255
Fax: (204) 789-3414

APPROVAL FORM

Principal Investigator: Dr. A. Katz

Ethics Reference Number: H2009:312
Date of REB Meeting: October 26, 2009
Date of Approval: November 9, 2009
Date of Expiry: October 26, 2010

Protocol Title: CIHR/CancerCare Manitoba Team in Primary Care Oncology Research Three Research Protocol: Innovative Tools to Improve Colorectal Cancer Screening Rates in Manitoba (Linked to H2007:141)

The following is/are approved for use:

- **Proposal, Version submitted October 9, 2009 with revisions outlined in correspondence dated November 9, 2009**
- **Research Participant Information and Consent Form – Control Group, Version 2.0 dated 01/11/09**
- **Research Participant Information and Consent Form – Intervention Group, Version 2.0 dated 01/11/09**
- **In Clinic Patient Survey, Version 2.0 dated 01/11/09**
- **Four Month Patient Survey – Control Group, Version 2.0 dated 01/11/09**
- **Four Month Patient Survey – Intervention Group, Version 2.0 dated 01/11/09**
- **Primary Care Provider Survey, Version 1.0 dated 01/11/09**
- **Primary Care Provider Recruitment Letter, Version 2.0 dated 01/11/09**
- **Primary Care Provider Response Letter, Version 2.0 dated 01/11/09**
- **Primary Care Provider Recruitment Reminder Letter, Version 1 dated October, 2009**
- **Primary Care Provider Clinic Characterization Form, Version 1 dated October, 2009**
- **Primary Care Provider Patient Tracking Form, Version 1 dated October, 2009**

The above was approved by Dr. John Arnett, Ph.D., C. Psych., Chair, Health Research Ethics Board, Bannatyne Campus, University of Manitoba on behalf of the committee per your letter dated November 9, 2009. The Research Ethics Board is organized and operates according to Health Canada/ICH Good Clinical Practices, Tri-Council Policy Statement, and the applicable laws and regulations of Manitoba. The membership of this Research Ethics Board complies with the membership requirements for Research Ethics Boards defined in Division 5 of the *Food and Drug Regulations of Canada*.

This approval is valid for one year from the date of the REB meeting at which the study was reviewed. A study status report must be submitted annually and must accompany your request for re-approval. Any significant changes of the protocol and informed consent form should be reported to the Chair for consideration in advance of implementation of such changes. The REB must be notified regarding discontinuation or study closure.

This approval is for the ethics of human use only. For the logistics of performing the study, approval must be sought from the relevant institution, if required.

Sincerely yours,

John Arnett, Ph.D., C. Psych.
Chair, Health Research Ethics Board
Bannatyne Campus

Please quote the above Ethics Reference Number on all correspondence.
Inquiries should be directed to the REB Secretary **Telephone**: (204) 789-3255 / Fax: (204) 789-3414
