# Supplementary material for: Does access to a colorectal cancer screening website and/or a nurse-managed telephone help line provided to patients by their family physician increase fecal occult blood test uptake?: A pragmatic cluster randomized controlled trial study protocol
Source: BMC Cancer. 2012 May 17;12:182. doi: 10.1186/1471-2407-12-182 (PMC3495851; doi:10.1186/1471-2407-12-182)
Supplement: Additional file 7 — Proof of Health Research Ethics Board (University of Manitoba) approval. University of Manitoba Health Research Ethics Board approval of Theme Three research study entitled “Innovative Tools to Improve Colorectal Cancer Screening in Manitoba” (H2009:312); Trial Registration: clinicaltrials.gov identifier NCT01026753. [file 1471-2407-12-182-S7.pdf]

UNIVERSITY OF MANITOBA

BANNATYNE CAMPUS
Research Ethics Boards

P126-770 Bannatyne Avenue
Winnipeg, Manitoba
Canada R3E 0W3
Tel: (204) 789-3255
Fax: (204) 789-3414

October 26, 2009

Dr. A. Katz
c/o K. Clouston
Family Medicine
P228 – 770 Bannatyne Avenue
Winnipeg, MB

Dear Dr. Katz:

Re: H2009:312
CIHR/CancerCare Manitoba Team in Primary Care Oncology Research Three Research Protocol: Innovative Tools to Improve Colorectal Cancer Screening Rates in Manitoba (Linked to H2007:141)

In response to your submission dated October 9, 2009 the above-named study was reviewed by the full board at the meeting of the Health Research Ethics Board on October 26, 2009 and will be considered for approval conditional to the following revisions:

- Please add to the confidentiality section of the Research Participant Information and Consent Form (ICF) the following statement "**The Health Research Ethics Board may review records related to this project for quality assurance purposes**".
- Please change "**Biomedical**" to "**Health**" Research Ethics Board in the ICF.

The issue(s) outlined must be met to the satisfaction of the chair of the Board prior to providing a certificate of final approval. When you have completed the requested revisions, **one (1) copy** of the revised portions of your project must be submitted to the Bannatyne Campus Research Ethics Office. **Please summarize your revisions and address the concerns of the board in a cover letter and make sure your revision is highlighted so they can be identified in the text of the revised document. Also, note the Health Research Ethics Board deadlines do not apply to the review of these revisions therefore you may submit them as soon as they are complete.**

Yours sincerely,

John Arnett, Ph.D., C. Psych
Chair, Health Research Ethics Board
Bannatyne Campus

JA/lw

Please quote the above Ethics Reference Number on all correspondence. Inquiries should be directed to the REB Coordinator. **Telephone**: (204) 789-3389/ **Fax**: (204)789-3414
