# Supplementary material for: Does access to a colorectal cancer screening website and/or a nurse-managed telephone help line provided to patients by their family physician increase fecal occult blood test uptake?: A pragmatic cluster randomized controlled trial study protocol
Source: BMC Cancer. 2012 May 17;12:182. doi: 10.1186/1471-2407-12-182 (PMC3495851; doi:10.1186/1471-2407-12-182)
Supplement: Additional file 8 — Proof of Health Research Ethics Board (University of Manitoba) approval. Final Health Research Ethics Board approval from the University of Manitoba Health Research Ethics Board. Approval of the theme three research study entitled “Innovative Tools to Improve Colorectal Cancer Screening in Manitoba” (H2009:312); Trial Registration: clinicaltrials.gov identifier NCT01026753. [file 1471-2407-12-182-S8.pdf]

April 3, 2007

Dr. KATZ, Alan
Manitoba Centre for Health Policy
4th Floor. Room 408
727 McDermot Ave
Winnipeg, Manitoba
Canada
R3E 3P5

Dear Dr. KATZ,

On behalf of the Canadian Institutes of Health Research (CIHR) Institute of Cancer Research and Cancer Care Manitoba I am pleased to inform you that your application entitled “Primary Care Oncology: New Emerging Team (PCO-NET)” submitted to the Team Grant: Access to Quality Cancer Care competition, has been approved.

As CIHR does not notify co-applicants by mail of the decision taken, we ask that you inform those individuals involved along with their research institutions (if different from your own) of the outcome of this application. Please note also, that if you have not already done so, you are required to inform CIHR of any applications submitted or funds received, to support this research during the review of this proposal. This is to avoid possible duplication of funding for research.

The CIHR Team Grant policy now requires that all team grant recipients (including CIHR Team Grants, New Emerging Team Grants, and Emerging Team Grants) adhere to special branding requirements in naming their teams as a condition of receiving funding. This policy was formerly a requirement of the Team Grants program only, but it was recently also extended to the New Emerging Team Grants program.

In this particular competition, the policy requires that team titles use the format, "CIHR Team in…" followed by a succinct description of the team's activities that focuses on the health problem being targeted through the research. Some examples of suitable names include: CIHR Team in Frailty and Aging; CIHR team in Blood Management Research. In cases where there is another major funding partner, a shared title would be considered; an acceptable format would be: "CIHR/(partner name) Team in (area of research)."

As per the policy, the Team name must be used in all communication and promotion relating to the Grant. The team must also agree to proper use of the Team name as well as the use of the CIHR (and applicable partner) logo(s) on appropriate communications materials such as brochures, letterhead, publications, presentations and media materials. Recognition guidelines, including instructions on logo use, will be provided to successful applicants as part of the approval package.

In order for CIHR to proceed with the release of funding, I require the following information no later than **April 16, 2007**:

1. a suggested title that meets CIHR Team Grants Communication Requirements; and

2. written documentation that confirms this title will be used in all materials mentioned above. This documentation must be signed by the Nominated Principal Investigator (NPI) and an official from the NPI's institution.

According to CIHR policy, the institution responsible for withholding funding from the researcher(s) until the required approvals for any research involving human subjects, animals or agents identified in the Guidelines on Laboratory Biosafety have been given.

If research trainees will be paid out of this grant, you are responsible for ensuring that CIHR's minimum stipend levels are adhered to. Trainees must not be paid less than the minimum stipend levels mandated by CIHR. To obtain information on these stipend levels, consult the sub-section "Compensation-Related Expenses" in the Tri-Agency (CIHR, NSERC & SSHRC) Financial Guide (http://www.nserc.ca/professors_e.asp?nav=profnav&lbi=f3#compensation).

CIHR has recently updated guidelines entitled "Guidelines for Human Pluripotent Stem Cell Research" (http://www.cihr-irsc.gc.ca/e/31488.html). If your application involves this type of research or if in the future your project evolves toward the use of these cells, could you please inform us as soon as possible so that your research can be reviewed by the Stem Cell Oversight Committee that is mandated to specifically oversee this research.

Copies of the reviewers' individual comments are enclosed.

Congratulations on your success in this competition.

Should you have any questions or need a translation of review material into the other official language of any of the enclosed reviews, please contact Jonathan Faulkner, Program Delivery Officer, by telephone at (613) 946-6568 or by e-mail at jfaulkner@cihr-irsc.gc.ca.

Sincerely,

Mohammed Hannan, PhD
Deputy Director, Knowledge Creation Programs Branch
Research Portfolio

/jf

cc. Dr. Phillip Branton, Scientific Director, ICR
Dr. J. Davie, Provincial Director, Research, CCM
Finance
